# Targeting amyotrophic lateral sclerosis by neutralizing seeding-competent TDP-43 in CSF

**DOI:** 10.1093/braincomms/fcad306

**Published:** 2023-11-03

**Authors:** Mickael Audrain, Anne-Laure Egesipe, Noémie Tentillier, Laure Font, Monisha Ratnam, Lorene Mottier, Mathieu Clavel, Morgan Le Roux-Bourdieu, Alexis Fenyi, Romain Ollier, Elodie Chevalier, Florence Guilhot, Aline Fuchs, Kasia Piorkowska, Becky Carlyle, Steven E Arnold, James D Berry, Ruth Luthi-Carter, Oskar Adolfsson, Andrea Pfeifer, Marie Kosco-Vilbois, Tamara Seredenina, Tariq Afroz

**Affiliations:** Research, AC Immune SA, 1015 Lausanne, Switzerland; Research, AC Immune SA, 1015 Lausanne, Switzerland; Research, AC Immune SA, 1015 Lausanne, Switzerland; Research, AC Immune SA, 1015 Lausanne, Switzerland; Research, AC Immune SA, 1015 Lausanne, Switzerland; Research, AC Immune SA, 1015 Lausanne, Switzerland; Research, AC Immune SA, 1015 Lausanne, Switzerland; Research, AC Immune SA, 1015 Lausanne, Switzerland; Research, AC Immune SA, 1015 Lausanne, Switzerland; Research, AC Immune SA, 1015 Lausanne, Switzerland; Research, AC Immune SA, 1015 Lausanne, Switzerland; Research, AC Immune SA, 1015 Lausanne, Switzerland; Research, AC Immune SA, 1015 Lausanne, Switzerland; Research, AC Immune SA, 1015 Lausanne, Switzerland; Department of Physiology, Anatomy and Genetics, University of Oxford, Oxford OX1 3PT, UK; Department of Neurology and the Massachusetts Alzheimer’s Disease Research Center, Massachusetts General Hospital, Harvard Medical School, Boston, MA 02129, USA; Sean M. Healey & AMG Center for ALS & the Neurological Clinical Research Institute, Massachusetts General Hospital, Harvard Medical School, Boston, MA 02114, USA; Research, AC Immune SA, 1015 Lausanne, Switzerland; Research, AC Immune SA, 1015 Lausanne, Switzerland; Research, AC Immune SA, 1015 Lausanne, Switzerland; Research, AC Immune SA, 1015 Lausanne, Switzerland; Research, AC Immune SA, 1015 Lausanne, Switzerland; Research, AC Immune SA, 1015 Lausanne, Switzerland

**Keywords:** ALS, TDP-43, RT-QuIC, immunotherapy, biomarker

## Abstract

In amyotrophic lateral sclerosis, a disease driven by abnormal transactive response DNA-binding protein of 43 kDa aggregation, CSF may contain pathological species of transactive response DNA-binding protein of 43 kDa contributing to the propagation of pathology and neuronal toxicity. These species, released in part by degenerating neurons, would act as a template for the aggregation of physiological protein contributing to the spread of pathology in the brain and spinal cord. In this study, a robust seed amplification assay was established to assess the presence of seeding-competent transactive response DNA-binding protein of 43 kDa species in CSF of apparently sporadic amyotrophic lateral sclerosis patients. These samples resulted in a significant acceleration of substrate aggregation differentiating the kinetics from healthy controls. In parallel, a second assay was developed to determine the level of target engagement that would be necessary to neutralize such species in human CSF by a therapeutic monoclonal antibody targeting transactive response DNA-binding protein of 43 kDa. For this, evaluation of the pharmacokinetic/pharmacodynamic effect for the monoclonal antibody, ACI-5891.9, *in vivo* and *in vitro* confirmed that a CSF concentration of ≍1100 ng/mL would be sufficient for sustained target saturation. Using this concentration in the seed amplification assay, ACI-5891.9 was able to neutralize the transactive response DNA-binding protein of 43 kDa pathogenic seeds derived from amyotrophic lateral sclerosis patient CSF. This translational work adds to the evidence of transmission of transactive response DNA-binding protein of 43 kDa pathology via CSF that could contribute to the non-contiguous pattern of clinical manifestations observed in amyotrophic lateral sclerosis and demonstrates the ability of a therapeutic monoclonal antibody to neutralize the toxic, extracellular seeding-competent transactive response DNA-binding protein of 43 kDa species in the CSF of apparently sporadic amyotrophic lateral sclerosis patients.

## Teaser

Audrain *et al*. report the detection of seeding-competent transactive response DNA-binding protein of 43 kDa (TDP-43) in CSF of amyotrophic lateral sclerosis patients using seed amplification assay. These seeding species can be neutralized by an antibody targeting the C-terminal of TDP-43 providing evidence for an immunotherapy approach to target amyotrophic lateral sclerosis.

## Graphical Abstract

**Graphical Abstract:**
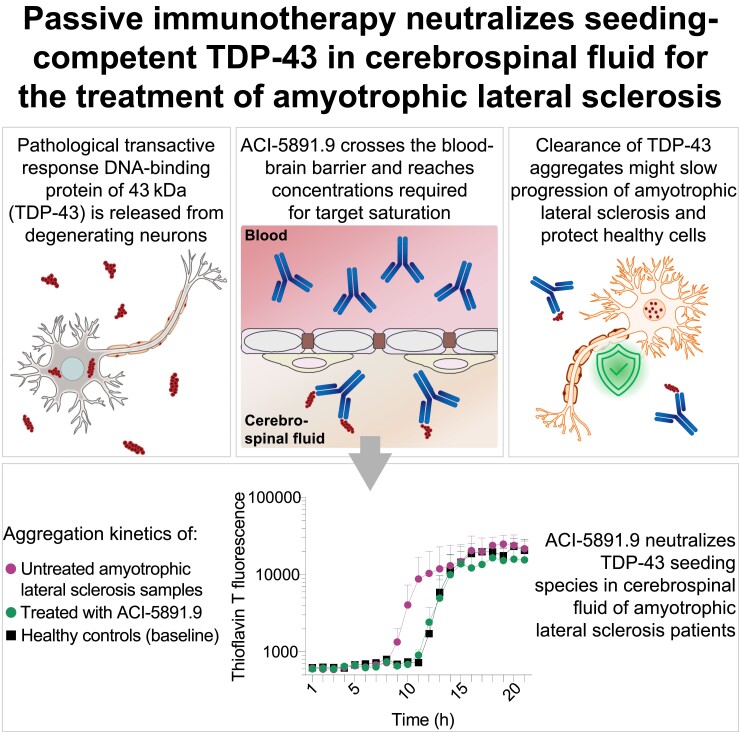

## Introduction

Amyotrophic lateral sclerosis (ALS) is a debilitating neurodegenerative disorder with high unmet medical need due to the lack of disease-modifying therapies.^1^ The disease primarily manifests in the CNS with the loss of upper and lower motor neurons leading to progressive motor dysfunctions.^1^ ALS is recognized as a proteinopathy where 97% of cases are characterized pathologically by ubiquitinated transactive response DNA-binding protein of 43 kDa (TDP-43) containing inclusions in affected neuronal and glial cells in the CNS.^2,3^ While TDP-43 is an essential nuclear protein regulating RNA metabolism, in pathological conditions, TDP-43 mislocalizes forming aggregates in the cytoplasm of neurons and glial cells.^2^ This not only leads to loss of TDP-43 function resulting in abnormal processing of essential neuronal RNA targets but also causes dysfunctions in mitochondria^4^ and nucleo-cytoplasmic transport^5^ due to toxic gain of function of TDP-43 aggregates. Staging of TDP-43 pathology based on post-mortem immunohistochemical analysis of ALS brain and spinal cord has revealed the spatio-temporal spread of pathology from the initially affected region to other neuroanatomically connected brain regions.^6,7^ These data advocate for the presence of specific pathogenic forms of TDP-43 that can transmit from cell to cell and transform conformation of the native protein into pathogenic form in a prion-like mechanism. The cell-to-cell spreading of TDP-43 pathology in brain has been demonstrated in transgenic mouse models injected with pathogenic TDP-43 seeds derived from brains of patients with TDP-43 proteinopathies.^8^ More recently, spinal cord extracts from ALS patients were demonstrated to seed and spread TDP-43 pathology in ALS cerebral organoids.^9^ However, this mechanism by itself does not explain the non-continuous clinical manifestations frequently seen in ALS.^10^ To account for this, it has been hypothesized that CSF can be a vector for local and distal propagation with subsequent disease progression occurring independently either via axonal^11^ transport (anterograde and/or retrograde), tunneling nanotubes,^12^ extracellular vesicles^13^ and CSF transmission.^14^ In fact, the neurotoxicity conferred by CSF of ALS patients *in vitro* suggests the presence of one or more toxic factors in ALS CSF,^15^ supporting an implication in disease pathogenesis.^16^ In ALS, a primary TDP-43 proteinopathy, it is hypothesized that disease-associated misfolded TDP-43 forms resulting from neurodegeneration in the CNS might be present in patient’s CSF and thereby contribute to disease pathogenesis.^10^

Several attempts have been made to establish the impact of ALS patient CSF exposure on TDP-43 aggregation and the associated downstream features *in vitro*^12^ and *in vivo*.^14,17^ Recently, an ultrasensitive assay based on amplification of seeds has emerged that enabled the detection of misfolded TDP-43 forms in CSF from confirmed genetic ALS patients that ‘seed’ or ‘induce’ aggregation of native TDP-43.^18^ Such a seed amplification assay (SAA) was originally conceptualized for *antemortem* detection of prions under the name of protein misfolding cyclic amplification and was based on the tendency of misfolded prion seeds to serve as template and induce native protein to shift to pathogenic conformation.^19^ Subsequently, the assay was successfully adapted to detect tau,^20-22^ α-synuclein^23-25^ and TDP-43^18^ seeds and has been reported under the name of real-time quaking-induced conversion. In this report, based on the recent consensus, such a technique is referred to as a SAA to reflect the proposal for a unifying terminology for this concept.^26^

To study the seeding propensity of CSF obtained from ALS patients and establish the translational potential to neutralize TDP-43 seeds with immunotherapy, the C-terminal region targeting TDP-43 monoclonal antibody (mAb), ACI-5891, was assessed. Previously, we have established that this region of TDP-43 is key for efficacy.^27^ The published data clearly demonstrated, using multiple *in vitro* and *in vivo* models of ALS and frontotemporal dementia (FTD), that mAbs such as ACI-5891 with epitopes in the C-terminal region result in the reduction of pathology as well as confer neuroprotection from toxic patient brain-derived TDP-43 aggregates.^27^ Furthermore, the sequence of ACI-5891 was humanized to a therapeutic suitable format, ACI-5891.9, which involved insightful antibody engineering, producing a candidate with optimal potency and pharmacokinetics to maximize successful disease modification in upcoming clinical trials.^28^

Thus, to study patient-derived extracellular seeding-competent TDP-43 and implications for clinical research, a robust SAA was established, employing a unique reaction substrate to both confirm the presence of seeding-competent TDP-43 species and determine the effect using a therapeutic mAb, ACI-5891.9. In parallel, a novel target engagement assay complemented with pharmacokinetic (PK) data from non-human primates (NHPs) was created to obtain the pivotal data to model the dose needed for target saturation in patients. Together, the data confirmed that CSF of apparently sporadic ALS patients contains species which can induce TDP-43 aggregation. Furthermore, when using the high-affinity mAb, ACI-5891.9, full inhibition of seed amplification was observed at clinically relevant concentrations. Hence, these data add to the growing body of literature to support that spreading of pathology in ALS occurs at least in part by species found in CSF. As such, treating patients with a passive immunotherapy approach by ACI-5891.9 should result in slowing and ameliorating devasting TDP-43-mediated neurodegenerative diseases such as ALS.

## Materials and methods

### Human CSF samples

Human CSF was provided by the Northeast Amyotrophic Lateral Sclerosis Biofluids Repository, Boston, USA. All samples were collected and used with written informed consent. Participants were not tested for all known ALS-causative genes, however, as these participants had no family history of ALS or FTD, they were classified as ‘apparently sporadic ALS’. Due to the small size of cohorts, the healthy controls (HCs) were not perfectly age matched, but majority of them overlapped with the ALS patients in age (Table 1). CSF was obtained by sterile lumbar puncture procedure by a qualified clinician, collected in sterile, polypropylene tubes, rapidly divided into aliquots, in polypropylene cryovials, and stored at − 80°C. The samples were handled without repeated freeze-thawing. Before measurements, samples were thawed at room temperature (RT) and vortexed.

**Table 1:** Summary of CSF samples used in SAA

CSF	Apparently sporadic ALS (sALS)	Healthy control (HC)
Number of donors	42	14
Age at collection [mean (SD), range]	58.8 (10.2), 39–78	41.0 (15.4), 19–59
Age at symptom onset [mean (SD), range]	55 (10.8), 27–74	
Disease duration at sample collection [mean (SD), range]	3.8 (3.2), 1–17	
Gender, female in %	45.2	42.9
Site of onset [limb, bulbar] in %	84, 16	
Ethnic category [non-Hispanic or Latino, Hispanic or Latino] in %	100, 0	86.7, 13.3
ALSFRS total score at first visit [mean (SD), range]	36.2 (6.5), 18–47	

ALSFRS, amyotrophic lateral sclerosis functional rating scale; SD, standard deviation.

### SAA substrate production

The TDP-43 peptide used as assay substrate (H-^352^NNQNQGNMQREPNQAFGSGNNSYSGSNSGAAIGWGSASNAGSGSGFNGGFGSSMDSKSSGWGM^414^-OH) was chemically synthesized at Pepscan and obtained with a purity >99% (UPLC/UV_215_). Lyophilized powder was first dissolved in pure hexafluoro-2-propanol (HFIP) for 5 days under agitation at RT and let dry under the hood overnight (200 µL of HFIP for 0.5 mg of lyophilized substrate). Films were then resuspended in ice-cold H_2_O, aliquoted and kept at −80°C. Concentration was assessed by measuring UV absorbance at 280 nm using NanoDrop Spectrophotometer (Thermo Fisher).

### TDP-43 SAA for CSF samples

**Figure 1:**
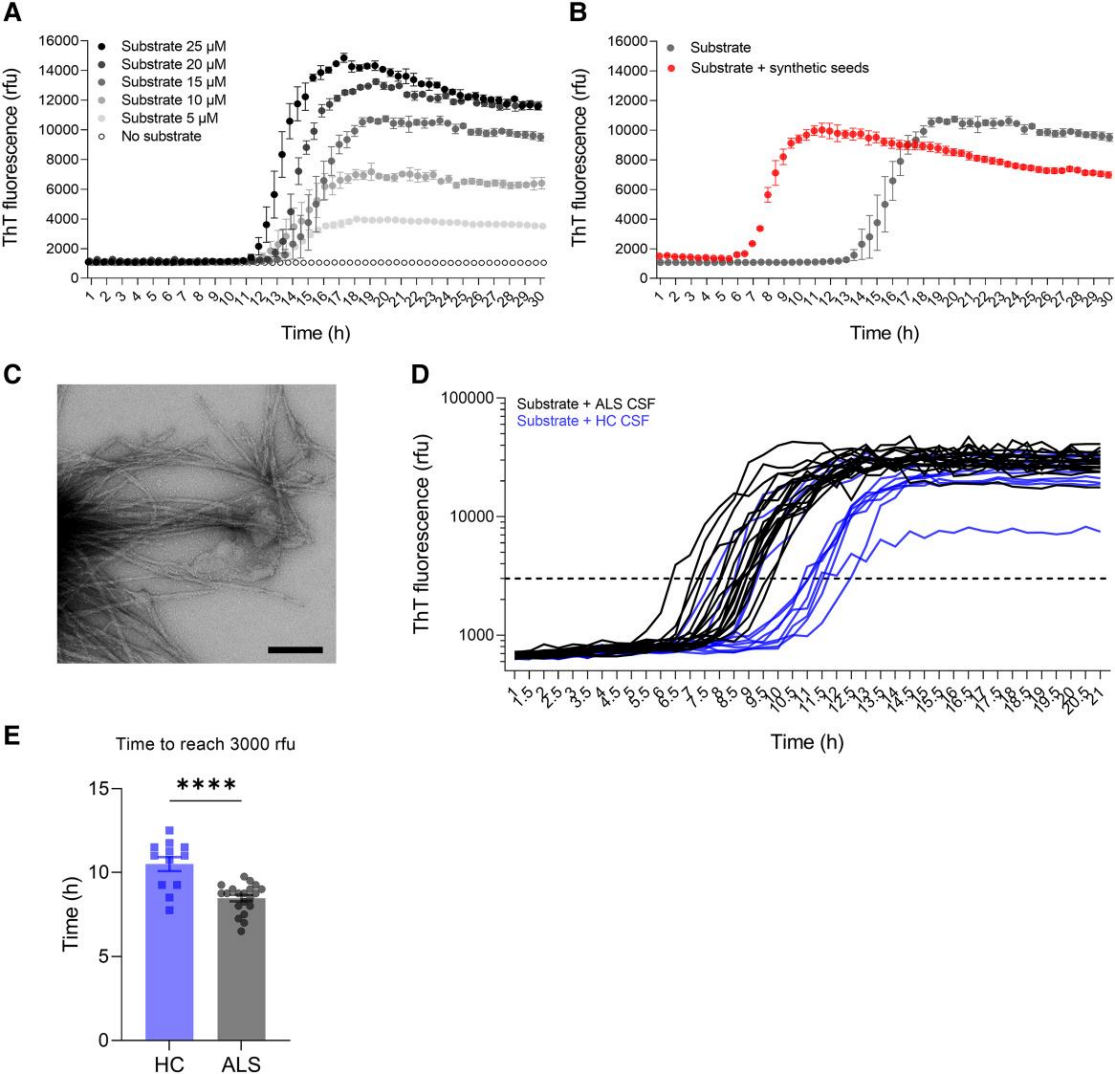
**Detection of seeding-competent extracellular TDP-43 in CSF of ALS patients.** (**A**) Aggregation kinetics of a seed-free TDP-43 peptide (352–414) used as the substrate for the SAA. Formation/accumulation of β-sheet-rich structures for a range of concentrations of substrate was evaluated over time by measuring the ThT fluorescence every 30 min. (**B**) Kinetics of a seed-free (grey) and seeded (red) aggregation where the final product of the 15 µM condition described in **A** was used as synthetic seeds. (**C**) Electron micrographs of assay substrate fibrils obtained after amplification reaction. Scale bar = 200 nm. (**D**) CSF from apparently sporadic ALS patients (*n* = 20, black) and age-matched HCs (*n* = 12, blue) were used as seeds in the SAA with 10 µM of substrate. An arbitrary amplification threshold was defined in the exponential phase at 3000 rfu (dotted line). (**E**) Quantification of the time to reach 3000 rfu. Data shown as mean ± standard error. Unpaired Student’s *t*-test (two-tailed), ****P < 0.0001.

Reactions were performed in 96-well black/clear bottom plates (Thermo Fisher #265301) in a total volume of 200 µL per well and in the presence of one glass bead of 3 mm diameter (Supelco #104015) in each well. The reaction master mix contained 40 mM Tris-HCl, 5 mM DTT, 0.002% SDS, 50 µM thioflavin T (ThT) and 10 µM substrate peptide (additional concentrations were tested in Fig. 1). Five microlitres of undiluted and unprocessed human CSF was used as seed per well. Plates were sealed and incubated at 40°C in a Tecan i-control (excitation/emission: 440/485 nm) with intermittent shaking cycles (amplitude 1.5 mm) of 1 min every 30 min. Two kinds of parameters were quantified: the time in hours needed to reach a certain value of fluorescence during the exponential phase of aggregation and the amount of fluorescence measured at different time points.

For seeding neutralization experiments, 1000 ng/mL of either ACI-5891.9 or IgG1 isotype control was preincubated with 5 µL undiluted and unprocessed CSF for 30 min at RT with agitation in the SAA master mix, without the substrate and ThT. These two reagents were added immediately prior to incubation at 40°C. Fluorescence (rfu) was arbitrarily compared at 12 h between the different conditions. Additionally, the difference in time to reach 3000 rfu between each sample incubated with ACI-5891.9 versus isotype control (ratio) was assessed.

### Electron microscopy to assess aggregation of assay substrate in SAA

The morphology of assay substrate assemblies was assessed by transmission electron microscopy in a CM 100 Biotwin transmission electron microscope (Philips) following adsorption onto formvar carbon-coated 400 mesh grids and negative staining with 1% uranyl acetate. The images were recorded using a bottom-mount TVIPS F416 camera (4K × 4K).

### Recombinant TDP-43 production

Human TDP-43 was expressed in *Escherichia coli* as a fusion protein with N-terminal His-SUMOstar tag. Protein expression was induced with 0.1 mM IPTG at 18°C for 16 h in Luria–Bertani medium (Sigma) supplemented with kanamycin. Following cell lysis, the protein was purified from the soluble fraction using affinity chromatography. Subsequently, the His-SUMOstar tag was removed using the SUMOstar Protease I (Tebu-bio). Finally, the protein was applied to size exclusion chromatography using 16/600 Superdex 75 column to obtain high-purity soluble protein in buffer (40 mM HEPES pH 8.0, 500 mM KCl, 20 mM MgCl_2_, 200 mM l-arginine, 5% glycerol, 0.05% NP-40, and 0.1 mM TCEP).

### Total TDP-43 quantifications in human CSF

The Single Molecule Array (SIMOA®; Quanterix Corporation, USA) technology was used for the development of high-sensitivity custom immunoassays using two proprietary murine IgG2a mAbs binding TDP-43: ACI-5891 and ACI-5965. ACI-5891 was conjugated to paramagnetic beads (Quanterix Corporation) as a capture reagent, and ACI-5965 was biotinylated (Thermo Fisher) to be used as a detection antibody. Assays were performed using a three-step protocol with all incubation steps performed at 25°C on a microplate shaker set at 800 rpm (Quanterix Corporation). The incubation times were 30 min with the antibody-conjugated capture beads, 10 min with the biotinylated detection antibody, and 10 min with streptavidin-β-d-galactosidase (SBG; Quanterix Corporation). Plates were washed between each incubation using a magnetic microplate washer (Quanterix Corporation) and read with a SR-X SIMOA® instrument (Quanterix Corporation). Assay optimizations were accomplished by adjusting the concentrations of mAbs either conjugated to the paramagnetic beads or after biotinylation. The molar ratio of biotin and linker used for mAb–biotin conjugations and the concentrations of SBG were also adjusted. Paramagnetic beads conjugated to the capture antibody were diluted in bead diluent (Quanterix Corporation), and human CSF samples were diluted in the general detector and sample diluent (Quanterix Corporation). SBG was diluted in SBG diluent (Quanterix Corporation). The lower limit of quantification was determined as the mean blank for a readout of average enzyme per bead (AEB) + 2.5 times the standard deviation. The lower limit of detection was set based on the signal at the lowest concentration of the calibrator (0.023 AEB). For calibrator, full length recombinant TDP-43 with a polyglycine tag in *N*-terminal was used (described above). The raw values in AEB rather than the extrapolated concentrations for CSF were used as the levels of TDP-43 signal was between lower limit of detection and lower limit of quantification.

### Free (unbound) TDP-43 quantifications in human CSF to determine ACI-5891.9 half-maximal inhibitory concentration

The assay described above was used to quantify free (unbound) TDP-43 in human CSF. Different concentrations (from 0 to 5000 ng/mL) of ACI-5891.9 (ACI-5891 humanized variant on human IgG1 backbone) were spiked into three different human CSF samples prior to use in the assay. This resulted in concentration-dependent saturation of the epitope by ACI-5891.9, preventing capture of TDP-43 by the ACI-5891-coated magnetic beads and allowing assessment of target engagement by measuring free TDP-43. A four-parameter logistic regression model was used to plot each curve for each human CSF sample independently.

### Humanized IgG1 antibody production

The Fv domain of ACI-5891 was discovered by hybridoma technology, from mice immunized using the SupraAntigen® vaccine technology.^28^ Murine complementarity-determining regions (CDRs) of ACI-5891 VH and VL were grafted onto selected frameworks VH1-69-2/VK2-28. Back-mutations were introduced at critical framework residues known to influence CDR conformation and based on the molecular model of ACI-5891 Fv domains to generate ACI-5891.9.^28^ For recombinant antibody productions, CHO cells were transiently transfected with equimolar quantities of heavy- and light-chain vectors. Antibodies were purified from supernatants by protein A chromatography (Cytiva, cat# 17543803). The identity and purity of the purified antibodies were confirmed using native and reducing SDS-PAGE. Concentrations of antibodies were measured using the NanoDrop Spectrophotometer (Thermo Fisher).

### NHP PK studies

The *in vivo* study was approved by the national ethical guidelines and performed in an animal facility accredited by the provincial animal management office. NHPs ≥ 2 years old, weighing ≥ 2 kg, were purchased from Hainan Jingang Laboratory Animal Co. Ltd. ACI-5891.9 was administered by intravenous bolus injection single dose at 40 mg/kg (*n* = 4). Serum was collected from each animal at the following time points: pre-dose (−24), 0.05, 1, 2, 4, 8, 16, 24, 48, 168, 336, 504, 672, 840, 1008, 1176 and 1344 h (Day 56).

Serum PK time points were selected to accurately capture the initial antibody concentration at the start of the study, the distribution phase during the first 48 h and the typical inflexion point of the PK profile (depicting a two-compartmental distribution behaviour expected for mAbs). Following this, weekly time points were collected for 8 weeks to characterize the elimination phase. PK parameters were estimated by non-linear mixed effect modelling using Monolix (2019R1, Lixoft) by a two-compartmental model with linear elimination from central compartment. PK parameters are reported as typical population PK parameters, and the terminal half-life was derived from it.

CSF was collected at the following time points: pre-dose (−24), 48 and 1344 h (Day 56), except for the NHP#1 for which both the pre-dose and 48 h time points were not collected as per protocol. The pre-dose time point allowed baseline quantification of TDP-43. The time point at 48 h post-dose allowed assessment of antibody penetration in the CNS when serum concentration was expected to be high, and the terminal time point was used to assess antibody concentration at the end of the study when antibody serum concentration had declined. Animals were checked daily for body condition, natural behaviour and appearance. Haematology and blood chemistry were performed pre-dose and at 168 h post-dose.

### Determination of ACI-5891.9 serum and CSF concentration in NHP PK study

The total concentration of human antibody in NHP serum was determined by enzyme-linked immunosorbent assay (ELISA). A biotinylated mouse polyclonal anti-human kappa chain antibody (Southern Biotech) diluted to 1.5 µg/mL in phosphate buffered saline (PBS) with 0.05% Tween 20 was captured onto a streptavidin-coated microplate (Microcoat Biotechnologie GmbH) for 1 h at RT. Plates were washed four times with PBS with 0.05% Tween 20 and blocked with 100 µL of PBS with 1% bovine serum albumin (BSA) and 0.05% Tween 20 (dilution buffer) for 1 h at RT. Standard curves prepared from a 2-fold serial dilution starting at 50 ng/mL and test samples diluted in 1:100 dilution buffer supplemented with 1% cynomolgus monkey serum (Neo-Biotech) were loaded to the plates, incubated for 90 min at RT and washed as previously described. Bound human antibodies were detected with an anti-human kappa chain antibody conjugated to horseradish peroxidase (Southern Biotech) diluted to 4.0 ng/mL in dilution buffer. Plates were incubated for 30 min at RT and washed as previously described. Finally, TMB substrate (SeraCare) was added to each well and incubated for 10–15 min. Reactions were stopped by adding 100 µL of Stop Solution (Bethyl) to each well. Absorbance was read at 450 nm with a reference wavelength at 690 nm using a BioTek microplate reader. Concentrations of all serum samples were back-calculated against a reference standard using a non-linear four-parameter regression fit including a 1/*y*^2^ weighting.

The total concentration of human antibody in NHP CSF was also determined by ELISA. Briefly, coating of 96-well plates was done with an anti-human IgG at 10 nM (=1.5 µg/mL) in carbonate–bicarbonate buffer (Sigma, SLBM9869V) using 50 µL/well at 4°C overnight. The washing steps were done using a plate washer and 200 µL per well of PBS/Tween 20 (0.05%). Blocking used 5% BSA in PBS/Tween 20 (0.05%) at RT for 30 min. Fifty microlitres per well of the sample (CSF, standards and quality controls) were incubated at 37°C for 1.5 h. CSF was diluted to 1/100. Incubation with the detection antibody, anti-human IgG F(ab’)_2_ (Jackson), at 20 nM (3 µg/mL) in PBS with 1% BSA and 0.05% Tween 20 for 1 h was performed at 37°C. Fifty microlitres per well of the TMB substrate (SeraCare) for 15 min at RT in the dark was used for the detection, and the reaction was stopped by adding 50 µL per well of H_2_SO_4_. Absorbance was read at 450 nm using a Tecan plate reader. Concentrations of CSF samples were also back-calculated using a reference standard using a non-linear four-parameter logistic model. The sensitivity of the assay was 0.93 ng/mL.

### Free TDP-43 quantifications to assess ACI-5891.9 target engagement in NHP serum

A similar assay design to that described for CSF was used to assess target engagement in the serum of NHPs previously treated with ACI-5891.9 during the PK study. However, capture and detection antibodies were inverted. The RNA recognition motif (RRM) binding TDP-43 mAb ACI-5965 was coupled to acceptor beads to capture total TDP-43, while detection was performed using a biotinylated ACI-5891. Additionally, serum samples were diluted (1/10) in a diluent (PBS, 300 mM NaCl, 2% BSA, 0.6% CHAPS, and 50 µg/mL TruBlock, pH 7.1) specifically formulated for optimum assay performance in serum. The concentration of free TDP-43 was extrapolated in picograms per millilitre or as a fold change compared to the pre-dose serum for each animal.

### Statistical analysis

Graphs represent the mean of all samples in each group ± SEM or SD. Sample sizes (*n* values) and statistical tests are also indicated in ﬁgure legends. For data in Fig. 1E and Supplementary Figs. 1D–G and 3C, an unpaired Student’s *t*-test (two-tailed) was used whereas for data in Fig. 3C, an ordinary one-way ANOVA followed by a Tukey’s test for *post hoc* analysis was used. The normal distribution of all data has been checked. *P*-values are reported as asterisks based on the following values: ****P < 0.0001, ***P < 0.001 and *P < 0.05.

## Results

### Seeding-competent TDP-43 in CSF of apparently sporadic ALS patients confirmed by SAA

To confirm the presence of seeding-competent TDP-43 in the CSF of apparently sporadic ALS patients, a SAA was developed. Amongst several TDP-43 peptides and proteins tested, one short peptide in the C-terminal region (amino acids 352–414) with characteristics suitable for SAA was identified. Following an initial lag phase, the substrate alone showed a concentration-dependent aggregation as monitored by ThT fluorescence, confirming the formation of β-sheet-containing fibrils (Fig. 1A). Following a rapid exponential phase, a plateau was reached for all tested concentrations demonstrating a consistent and reproducible reaction equilibrium (Fig. 1A). Importantly, when the seeds obtained at the end of the reaction were introduced at the beginning of a new reaction, an acceleration of the aggregation kinetics was observed (Fig. 1B). These data confirmed that the introduced seeds served as template to induce an acceleration of the aggregation of the monomeric substrate, validating the principle of the SAA for TDP-43. Ultrastructural evaluation of the aggregation products at the end of the reaction confirmed the presence of fibrils demonstrating the ability of this peptide to form β-sheet-rich fibrillar structures (Fig. 1C). The buffer conditions were further optimized to reduce the initially observed inter-experimental variability in substrate aggregation (Supplementary Fig. 1A). Solubilization of peptide in HFIP resulted in a consistent substrate aggregation from different batches of peptide preparations (Supplementary Fig. 1B).

Following the assay qualification, CSF from 20 apparently sporadic ALS patients and 12 HC (described in Table 1) were used as seeds in the SAA, and aggregation kinetics of the substrate was measured. CSF from apparently sporadic ALS patients accelerated the aggregation kinetics of the reaction substrate compared to HC, demonstrating the presence of seeding-competent TDP-43 in ALS CSF (Fig. 1D, Supplementary Fig. 1C). Time to reach an arbitrary fluorescence level (3000 rfu), which was chosen as in the exponential amplification phase, was significantly shorter in apparently sporadic ALS CSF (8.47 h) as compared to HC (10.50 h; Fig. 1E). Fluorescence values at various time points also showed significantly higher values in apparently sporadic ALS samples compared to HC (Supplementary Fig. 1D–F). The substrate aggregation kinetics observed in CSF from HC reflected the expected intrinsic aggregation of the assay substrate. Importantly, the optimized SAA differentiated the CSF obtained from apparently sporadic ALS patients versus HC in contrast to the assay measuring only the levels of total TDP-43 where equivalent levels of TDP-43 were measured in the two groups (Supplementary Fig. 1G).

### Determination of the pharmacologically relevant anti-TDP-43 mAb concentration in human CSF

**Figure 2:**
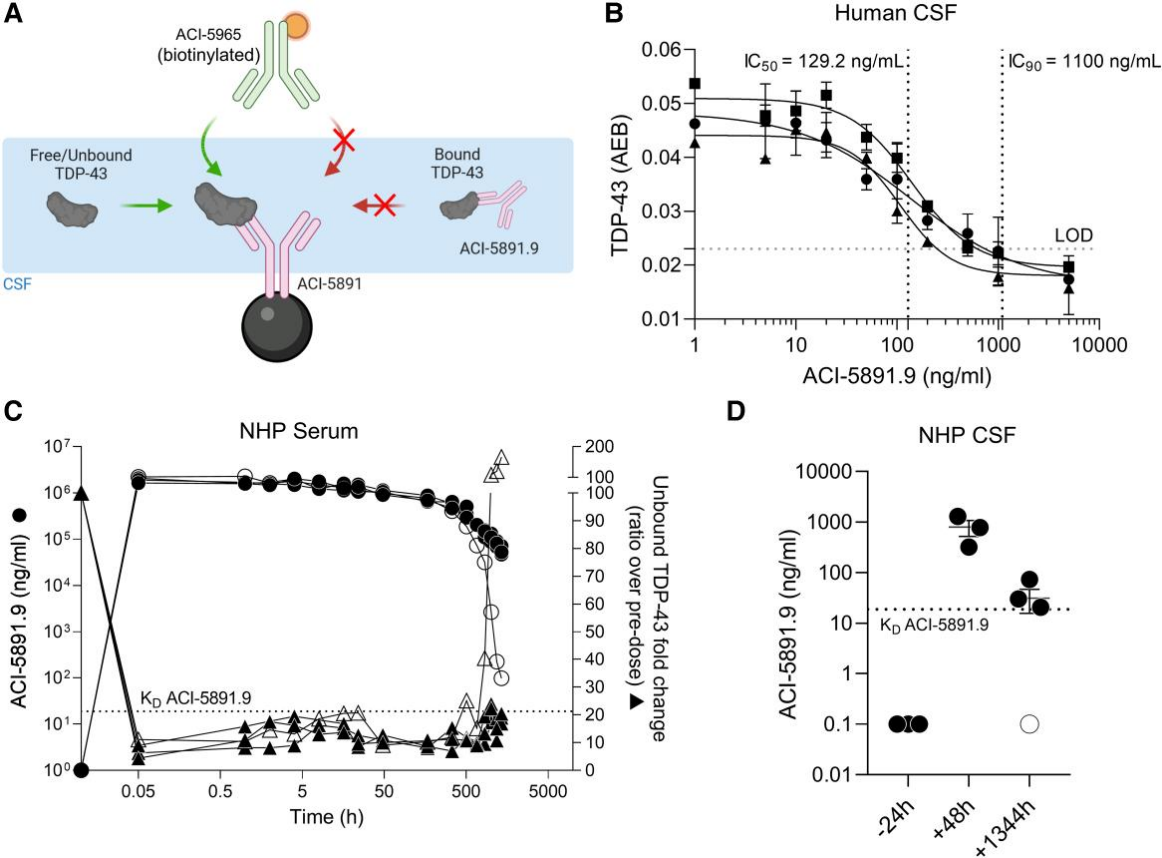
**Pharmacologically relevant concentrations to saturate TDP-43 in human CSF by ACI-5891.9 achieved *in vivo*.** (**A**) A SIMOA®-based assay to assess target engagement in CSF of NHPs (used for **B**). ACI-5891 was used as a capture antibody after coating to magnetic beads. A biotinylated version of ACI-5965 was used as a detection antibody. Arrows on the left indicate the ability of the assay to measure free TDP-43 as opposed to unmeasured immune complexes (shown by arrows on the right). (**B**) ACI-5891.9 IC_50_ estimation in human CSF using the assay described in **A**. Free TDP-43 signal shown on *y*-axis by measuring AEB in human CSF (*n* = 3) with increasing concentration of ACI-5891.9 (*x*-axis). (**C**) ACI-5891.9 PK/PD relationship in NHPs (*n* = 4) administered with ACI-5891.9 (single intravenous dose at 40 mg/kg). Left and right *y*-axes represent ACI-5891.9 exposure (circles) and free TDP-43 fold change (triangles), respectively. NHP#1 (open circles and triangles) presented ADA response. The dotted line represents ACI-5891.9 *K*_D_ (126 pM or 18.9 ng/mL). (**D**) ACI-5891.9 exposure in CSF of the same four NHPs (described in **C**) following single-dose intravenous administration at 40 mg/kg. Open circle in 1344 h time point indicates the animal with ADA.

The presence of TDP-43 seeding-competent species in CSF of ALS patients reinforces the rational for TDP-43 immunotherapy, using, for example, the high-affinity TDP-43 mAb, ACI-5891.^28^ To test whether the humanized mAb ACI-5891.9 could prevent seeding in ALS CSF samples, target binding and mAb concentration required to saturate TDP-43 in the CSF was evaluated. For this, a novel immunoassay using single-molecule array (SIMOA®) technology was set up. A mouse anti-human C-terminal targeting, TDP-43 mAb, ACI-5891, was coupled to acceptor beads allowing capture of total TDP-43 in human CSF. However, when ACI-5891.9, a humanized version of ACI-5891, was pre-incubated in human CSF sample, immune complexes with TDP-43 were formed impeding their capture in the assay due to the unavailability of the ACI-5891 epitope (Fig. 2A, Supplementary Fig. 2A). Therefore, only free (unbound) TDP-43 was measured using a non-competing TDP-43 detection mAb, ACI-5965, that bound in the RRMs (Fig. 2A, Supplementary Fig. 2A). Incubating with increasing concentration of ACI-5891.9 in human CSF resulted in a concentration-dependent decrease in free TDP-43 signal compared to the isotype control, demonstrating the specificity of the assay to quantify free TDP-43 in CSF (Supplementary Fig. 2B). When replicated in three additional human CSF samples (described in Table 2), an average half-maximal inhibitory concentration (IC_50_) of 129.2 ng/mL was calculated for ACI-5891.9 confirming its high binding affinity to TDP-43 (Fig. 2B). Importantly, based on IC_90_ (1100 ng/mL), these data demonstrated that a mAb concentration of approximately ≍1100 ng/mL was required to saturate all forms of TDP-43 in human CSF (Fig. 2B).

**Table 2:** Summary of CSF samples used to determine ACI-5891.9 IC_90_

CSF	Number of donors	Age	Gender
Dementia AD mixed	1	79	Male
FTD, behavioural variant	1	72	Female
Normal pressure hydrocephalus	1	77	Male

AD, alzheimer's disease; FTD, frontotemporal dementia.

### ACI-5891.9 CSF levels required for target saturation achieved *in vivo*

Having estimated the concentrations of ACI-5891.9 required for target saturation in human CSF, exposure levels were determined in CSF following peripheral administration of ACI-5891.9 in NHPs. To this end, a PK study was performed in which ACI-5891.9 was administered as a single dose of 40 mg/kg intravenously. ACI-5891.9 demonstrated excellent pharmacokinetics with a low clearance 0.083 mL/h/kg, resulting in a sustained mAb exposure with a half-life of 12.7 days (Fig. 2C, Supplementary Table 1 and Fig. 2C). No adverse clinical findings were observed in animals dosed with ACI-5891.9. Following confirmation of mAb exposure in serum, mAb levels in NHP CSF from the same study were quantified with an ELISA utilizing an anti-human IgG as capture antibody and an anti-human IgG F(ab’)_2_ detection antibody (Fig. 2D, Supplementary Fig. 2D).

However, due to the small number of animals in the study, only a limited number of time points were measured. Levels of up to 1000 ng/mL of ACI-5891.9, required for target saturation, were achieved in CSF 48 h after dose administration (Fig. 2D). Moreover, the low serum mAb clearance also resulted in a prolonged CSF exposure. Even at the terminal points (Day 56), CSF levels were close to the *K*_D_ of ACI-5891.9 (Fig. 2D). Taken together, these data demonstrated that ACI-5891.9, when administered peripherally, crossed the blood–brain barrier (BBB) to reach concentrations in the CNS required for sustained target saturation.

### ACI-5891.9 pharmacodynamics *in vivo*

To assess the pharmacodynamics of ACI-5891.9 *in vivo*, a second immunoassay using SIMOA® technology was developed. Briefly, the RRM binding TDP-43 mAb ACI-5965 was coupled to acceptor beads to capture total TDP-43, while detection was performed using a biotinylated ACI-5891. Similar to the assay principle described before, the formation of immune complexes by ACI-5891.9 *in vivo* precluded their detection by this assay due to the unavailability of ACI-5891 epitope (Supplementary Fig. 2E). This was indeed confirmed by spiking various concentrations of ACI-5891.9 in NHP serum prior to TDP-43 measurements in the assay (Supplementary Fig. 2F). As predicted, compared to the isotype control mAb, no TDP-43 signal could be measured with spiked ACI-5891.9 concentrations between 10 and 1000 µg/mL, demonstrating complete saturation of the target (Supplementary Fig. 2F). As the ACI-5891.9 serum concentrations in the PK study were in this range, this assay could be successfully used to measure target engagement in NHP serum following 40 mg/kg single-dose administration. Total TDP-43 levels measured at the baseline (pre-dose time point −24 h) were completely bound by ACI-5891.9 in the serum at all time points post-dosing, demonstrating efficient binding and formation of stable immune complexes (Fig. 2C, Supplementary Fig. 2G). This was consistent with the fact that serum mAb concentrations were at least three log units higher than the *K*_D_ of ACI-5891.9 at all time points up to 56 days, and complete target saturation was expected. Indeed, this was observed as the target remained bound until the final time points (Fig. 2C, Supplementary Fig. 2G), except in one animal where anti-drug antibodies (ADA) reduced the mAb concentrations at the later time points and a corresponding increase in the free TDP-43 was observed (Fig. 2C, Supplementary Fig. 2C and G), demonstrating the robustness and sensitivity of the assay to measure free TDP-43.

### ACI-5891.9 neutralizes pathogenic TDP-43 seeds in CSF obtained from apparently sporadic ALS patients at pharmacologically relevant concentration

**Figure 3:**
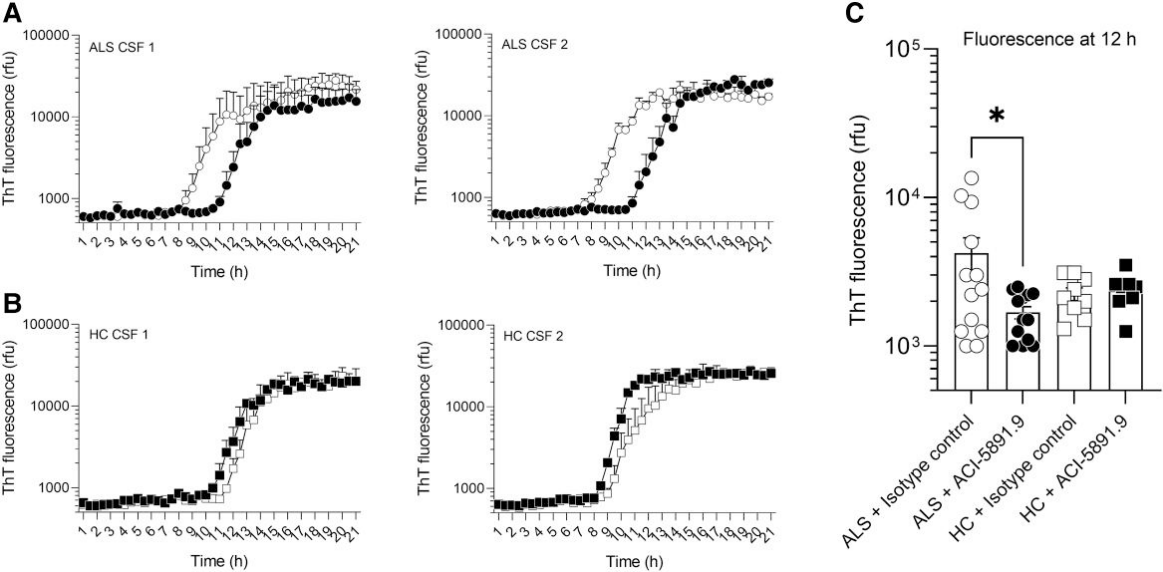
**ACI-5891.9 neutralizes TDP-43 seeding species in CSF of ALS patients.** (**A**) Apparently sporadic ALS patients or HCs. (**B**) CSF were pre-incubated for 30 min at RT with 1000 ng/mL of ACI-5891.9 (filled symbols) or 1000 ng/mL of the isotype control antibody (open symbols) prior to be used as seeds in the SAA. (**C**) Quantification of the fluorescence at 12 h. Data shown as mean ± standard error, for ALS (*n* = 13) and HC (*n* = 9). An ordinary one-way ANOVA followed by a Tukey’s test for *post hoc* analysis, *P < 0.05.

To test whether ACI-5891.9 can block TDP-43 seeding in patient CSF, the SAA was performed in the presence of the antibody. Briefly, the assay was performed after pre-incubating CSF of either ALS or HC with ACI-5891.9 or an isotype control mAb at a concentration of 1000 ng/mL (Supplementary Fig. 3A). This concentration close to the EC_90_ was able to saturate the target in human CSF (Fig. 2B), and such CSF mAb concentrations were achieved in NHPs following single dose at 40 mg/kg (Fig. 2D). Of note, even though the assay substrate contained the binding epitope of ACI-5891.9, the low mAb concentration (1000 ng/mL, i.e. 0.006 µM) as compared with the assay substrate (10 µM) did not affect the intrinsic aggregation of the SAA substrate *per se* (Supplementary Fig. 3B). Strikingly, incubating ACI-5891.9 with the CSF obtained from ALS patients delayed the aggregation of the substrate as compared to the isotype control (Fig. 3A). The isotype control had no effect, resulting in the expected faster aggregation in CSF of ALS as compared with HC (Fig. 3B). Furthermore, as no seeds were expected in the HCs, there was no effect of either ACI-5891.9 or the isotype control on the aggregation kinetics reflecting only the intrinsic aggregation of the substrate (Fig. 3B). By testing this in a larger number of CSF samples obtained from ALS patients and HC, a significant decrease in the measured fluorescence at the 12 h time point (in the exponential phase) was observed in CSF of ALS patients upon ACI-5891.9 incubation as compared to the isotype control, demonstrating neutralization and inhibition of seeding activity by ACI-5891.9 (Fig. 3C). As expected, there was no effect of ACI-5891.9 in any of the CSF samples from HC (Fig. 3C). In addition to reduction in aggregation, there was a delay in the aggregation kinetics in samples from ALS patients as compared to HC shown by the time required to reach an arbitrary fluorescence in the exponential phase when normalized by isotype control (Supplementary Fig. 3C). However, in some of the CSF samples from ALS patients, there was an unusual delay in the aggregation kinetics of the substrate in the presence of the isotype control compared to other samples and may represent outliers due to the normalization method used, especially as this phenomenon was also observed in a sample from the HC group (Supplementary Fig. 3C).

## Discussion

The existence of various pathological TDP-43 species released by degenerating neurons remains an attractive hypothesis to explain a number of clinical findings, including non-continuous manifestations observed particularly in ALS.^10^ Here, we confirm the presence of seeding-competent TDP-43 in the CSF from patients with apparently sporadic ALS similar to what was previously reported for genetic ALS cases.^18^ While the total amount of TDP-43 in both sets of CSF samples was equivalent, setting up an optimized SAA demonstrated the presence of seeding-competent species only in the CSF of apparently sporadic ALS patients. This finding is particularly clinically relevant as 90% of ALS cases are sporadic.^29^ Establishing the existence of such species next allowed us to prove that an anti-TDP-43 mAb, such as ACI-5891.9, would neutralize this pathological activity when provided in sufficient quantities. Translating to the clinical setting, we have modelled the *in vitro* and *in vivo* PK and pharmacodynamic data to support a feasible dose level administered systemically that will result in a concentration sufficient for target saturation in patients.

To date, the lack of knowledge concerning the nature of disease-mediating species of TDP-43 has been hampered by the paucity of fluid-based biomarker assays for TDP-43.^30^ Therefore, inconsistent results for total TDP-43 levels in the CSF of ALS and FTD patients have been reported.^30^ Moreover, the abundance of species carrying disease-specific post-translational modifications such as phosphorylation appears to be at the limit of detection of currently available methods.^30^ Unfortunately, the underlying limitation of these assays is their inability to detect the misfolded and/or aggregated species associated with disease that are hypothesized to induce aggregation of native protein in a prion-like manner.^31^ As the abundance of TDP-43 in CSF is low,^32^ the levels of misfolded TDP-43 can be below the detection levels of the current assays that aim to quantify TDP-43. Moreover, the seeding-competent species could also be specific truncated forms of TDP-43 harbouring the C-terminal amyloid region of TDP-43 as recently demonstrated by structural analysis.^33^ However, due to the unavailability of assays to specifically quantify those fragments, their identification and detection in CSF have not been possible.

Therefore, we set out to address these gaps by establishing a SAA in which we focused efforts on the amplification step to detect misfolded conformations that are present in CSF of patients. Prior research has suggested that seeding can be observed with the use of amplification in the CSF of patients with genetic forms of ALS such as *c9orf72*, *TARDBP* and *GRN*,^18^ which are associated with TDP-43 proteinopathy. Here, we developed the SAA to be sensitive enough to reproducibly detect such species in samples from apparently sporadic ALS patients. To achieve such an assay, several parameters were assessed. Importantly, the use of a short, C-terminal TDP-43 peptide as substrate generated the highest level of reproducibility in terms of substrate aggregation kinetics. Batch-to-batch variability in aggregation kinetics was optimized by pre-dissolving the substrate in HFIP that is known to disrupt pre-formed β-sheet structures.^34^ In combination with reproducible and consistent synthesis of substrate batches, this resulted in reproducible aggregation kinetics of the assay substrate. Moreover, the assay substrate overlapped partially with the C-terminal region employed in previously reported SAA^18^ and may explain the consistent results obtained in our assay for the detection of seeding species in CSF.

As with all novel biological assays, the aim is to share the current format with the scientific community and then continue to optimize with larger sample sets. The data obtained from samples shared by biobanks and consortiums will provide a better definition of assay parameters including the sensitivity and dynamic range as well as understanding the relevance for different patient subtypes. With larger and more diverse data sets, we envision clearer insights into, for example, how to meaningfully normalize results and set thresholds for samples that may present with a different profile (Supplementary Fig. 3C). The profile in four ALS and one HC samples in our small data pool was attributed to delayed aggregation kinetics of the assay substrate in the presence of the isotype control. We will test the hypothesis that as TDP-43 is an aggregation-prone protein, non-specific interactions between the isotype control and TDP-43 in these samples might result in such an altered aggregation profile.

The field advances with having the SAA methodology as described here as ultimately amplifying TDP-43 will be of use as both a diagnostic and pharmacodynamic readout in clinical trials when assessing modalities directly and indirectly targeting TDP-43. First, the SAA can be used to assist in pre-selection of ALS patients with confirmed seeding-competent TDP-43 in clinical trials evaluating TDP-43 targeting compounds. The current SAA can serve as a tool to screen ALS patients to confirm the presence of TDP-43 seeds in the CSF and exclude patients without TDP-43 pathology such as those with SOD1 or FUS pathology. Second, together with pre-clinical study data, the SAA can be used in the modelling to establish and support the choices for clinical doses. For example, the data from the SAA provide pharmacologically effective concentrations of the antibody required in the CSF of patients. Additionally, the SAA can be used as a proof of target engagement. As demonstrated in Fig. 3, the aggregation kinetics in CSF of patients returned to baseline level in the presence of saturating amounts of mAbs due to efficient neutralization of seeding species. Therefore, for pharmacodynamic biomarker evaluation, the CSF can be collected before and after the treatment with drugs targeting TDP-43 to evaluate whether the fast aggregation kinetics in pre-dose CSF returned to baseline levels to confirm efficient neutralization of seeding species. In addition, this assay might be valuable for diagnostics of other TDP-43 proteinopathies such as frontotemporal dementia, Alzheimer’s disease with TDP-43 pathology and limbic-predominant age-related TDP-43 encephalopathy, and future studies will explore this potential. Finally, additional studies will be required to evaluate longitudinal samples to determine the utility of this assay to monitor disease progression.

To further support the pharmacodynamic evaluation of passive immunotherapy using ACI-5891.9, a novel immunoassay was developed to quantify free TDP-43. This assay relied on the formation of stable immune complexes due to high affinity and low off-rate of the antibody for the target. This assay was used as a surrogate for binding to pathogenic TDP-43 as ACI-5891.9 binds to both physiological and pathological TDP-43 with high affinity. Such assays using binding to physiological protein as a surrogate for target engagement have been used for other therapeutic mAbs, e.g. an anti-α-synuclein mAb.^35,36^ Indeed, this setup can be used in future clinical trials to evaluate target engagement of ACI-5891.9. The PK assessment of ACI-5891.9 in NHP demonstrated prolonged exposure resulting in a robust target saturation in serum further confirming ACI-5891.9 potency *in vivo*. Interestingly, the level of TDP-43 quantified in NHP serum is very similar to that described for human plasma.^32^ Thus, a similar target saturation can be expected when conducting clinical studies.

In neurodegenerative disorders, CSF serves as a surrogate compartment for the CNS and mAbs have been demonstrated to cross the BBB upon systemic administration in clinical studies. The antibody concentrations in CSF have been reported in the range of 0.1% as compared to levels in the plasma.^37^ In addition, the BBB integrity in ALS could further enhance the distribution of drugs in the CNS of ALS patients.^38,39^ As this therapeutic antibody targeting TDP-43 may be the first in ALS clinical trial, this research has been focused on having assays that help determine the PK/PD relationships in patients to help define the safe dose needed for larger Phase 2 and 3 trials. For ACI-5891.9, levels up to 1000 ng/mL were attained in the CSF of NHPs at 48 h following single-dose administration at 40 mg/kg resulting in ≍90% target saturation based on the IC_90_. Moreover, based on the serum levels and assuming 0.1% brain penetration for a mAb,^37^ levels up to 200 ng/mL would be predicted in CSF of NHPs even after 1 month following single administration at 40 mg/kg. This value would be expected to be even higher in humans considering the expected longer half-life for human IgG1 in humans as compared to NHPs.^40^ Recent examples of successful target engagement for mAbs targeting amyloid beta in Alzheimer’s disease prove that therapeutically relevant levels of mAb can be achieved in the CNS of patients.^41^

Antibody pharmacodynamics was confirmed in the SAA with the use of a specific mAb ACI-5891.9 that does not cross-react with other prion-like proteins^28^ such as β-amyloid, α-synuclein or tau implicated in neurodegeneration.^42^ As the co-pathology of these proteins is commonly found in neurodegenerative diseases, their presence theoretically could influence the seeding observed in the SAA. However, this is not the case as seeding activity was neutralized in with ACI-5891.9, which is highly selective for TDP-43 and not cross-reactive to these or other proteins. These results reinforce the use of a TDP-43 mAb to prevent the propagation of pathology in ALS. Along those lines, ACI-5891 has been previously reported to mitigate TDP-43 aggregation and provide neuroprotection in *in vitro* and *in vivo* models of TDP-43 proteinopathies without altering the intracellular functions of TDP-43.^28^ This study expands the understanding of the mode of action of the humanized version of ACI-5891 in the patient CNS compartment to capture and clear the seeding species. Importantly for translation, the assay setup closely recapitulates the situation in patients where the CSF will be exposed to ACI-5891.9 following systemic administration. The formation of immune-complexes with TDP-43 seeds would further potentiate their clearance by the potent mechanism of Fc-gamma-mediated phagocytosis followed by degradation in microglia. Neuronal uptake, if any, would represent only a small fraction of the clearance mechanism.

To maximize the chances of capture, neutralization and clearance of all seeding-potent forms of TDP-43 including the C-terminal fragments found to be enriched in TDP-43 aggregates found in patients,^2^ ACI-5891.9 targeting the C-terminal low complexity domain was essential. The intrinsic propensity of the C-terminal region of TDP-43 to form higher-order assemblies allows the formation of physiological structures such as stress granules.^43^ However, in disease state, irreversible inter- and intra-molecular interactions could occur within this region giving rise to pathologic protein aggregates.^43^ Three independent studies^42,44,45^ have demonstrated that an immunotherapy approach could be beneficial to target TDP-43 in disease and that targeting the C-terminal region of TDP-43 would be essential to achieve efficacy.^42,45^ Even though a specific part of the C-terminal region (amino acids 280–360) has been shown to represent the amyloid core of pathological TDP-43 aggregates in the brain of ALS patients,^33,46^ regions adjacent to this core have been demonstrated to adopt β-sheet conformation.^47^ This was further demonstrated in this study by the ThT-positive fibrillar structure adopted by the assay substrate (amino acids 350–414).

In conclusion, in addition to providing the field with an optimized SAA, this study provides evidence for extracellular seeding-competent disease-associated TDP-43 species in the CSF of patients with apparently sporadic ALS. The extensive work to determine saturating conditions both *in vitro* and *in vivo* for use of an immunotherapy approach with ACI-5891.9 can be used in support of clinical rational and dosing regimens for trials in patients with apparently sporadic ALS. Furthermore, the SAA and target engagement assays can be used to help stratify and follow efficacy of disease-modifying therapies in patients.

## Supplementary Material

fcad306_Supplementary_Data

## Data Availability

All data associated with this study are in the main manuscript or in the Supplementary material.

## References

[1] Brown RH Jr, Al-Chalabi A. Amyotrophic lateral sclerosis. N Engl J Med. 2017;377(16):1602. 10.1056/NEJMc1710379 29045202

[2] Neumann M, Sampathu DM, Kwong LK, et al Ubiquitinated TDP-43 in frontotemporal lobar degeneration and amyotrophic lateral sclerosis. Science. 2006;314(5796):130–133. 17023659 10.1126/science.1134108

[3] Ling SC, Polymenidou M, Cleveland DW. Converging mechanisms in ALS and FTD: Disrupted RNA and protein homeostasis. Neuron. 2013;79(3):416–438. 23931993 10.1016/j.neuron.2013.07.033 PMC4411085

[4] Wang P, Deng J, Dong J, et al TDP-43 induces mitochondrial damage and activates the mitochondrial unfolded protein response. PLoS Genet. 2019;15(5):e1007947. 31100073 10.1371/journal.pgen.1007947 PMC6524796

[5] Chou CC, Zhang Y, Umoh ME, et al TDP-43 pathology disrupts nuclear pore complexes and nucleocytoplasmic transport in ALS/FTD. Nat Neurosci. 2018;21(2):228–239. 29311743 10.1038/s41593-017-0047-3 PMC5800968

[6] Brettschneider J, Del Tredici K, Toledo JB, et al Stages of pTDP-43 pathology in amyotrophic lateral sclerosis. Ann Neurol. 2013;74(1):20–38. 23686809 10.1002/ana.23937 PMC3785076

[7] Young AL, Vogel JW, Robinson JL, et al Data-driven neuropathological staging and subtyping of TDP-43 proteinopathies. Brain. 2023;146(7):2975–2988. 37150879 10.1093/brain/awad145 PMC10317181

[8] Porta S, Xu Y, Restrepo CR, et al Patient-derived frontotemporal lobar degeneration brain extracts induce formation and spreading of TDP-43 pathology in vivo. Nat Commun. 2018;9(1):4220. 30310141 10.1038/s41467-018-06548-9 PMC6181940

[9] Tamaki Y, Ross JP, Alipour P, et al Spinal cord extracts of amyotrophic lateral sclerosis spread TDP-43 pathology in cerebral organoids. PLoS Genet. 2023;19(2):e1010606. 36745687 10.1371/journal.pgen.1010606 PMC9934440

[10] Smith R, Myers K, Ravits J, Bowser R. Amyotrophic lateral sclerosis: Is the spinal fluid pathway involved in seeding and spread? Med Hypotheses. 2015;85(5):576–583. 26220261 10.1016/j.mehy.2015.07.014

[11] Feiler MS, Strobel B, Freischmidt A, et al TDP-43 is intercellularly transmitted across axon terminals. J Cell Biol. 2015;211(4):897–911. 26598621 10.1083/jcb.201504057 PMC4657165

[12] Ding X, Ma M, Teng J, et al Exposure to ALS-FTD-CSF generates TDP-43 aggregates in glioblastoma cells through exosomes and TNTs-like structure. Oncotarget. 2015;6(27):24178–24191. 26172304 10.18632/oncotarget.4680 PMC4695178

[13] Gagliardi D, Bresolin N, Comi GP, Corti S. Extracellular vesicles and amyotrophic lateral sclerosis: From misfolded protein vehicles to promising clinical biomarkers. Cell Mol Life Sci. 2021;78(2):561–572. 32803397 10.1007/s00018-020-03619-3 PMC7872995

[14] Mishra PS, Boutej H, Soucy G, et al Transmission of ALS pathogenesis by the cerebrospinal fluid. Acta Neuropathol Commun. 2020;8(1):65. 32381112 10.1186/s40478-020-00943-4 PMC7206749

[15] Ng Kee Kwong KC, Gregory JM, Pal S, Chandran S, Mehta AR. Cerebrospinal fluid cytotoxicity in amyotrophic lateral sclerosis: A systematic review of in vitro studies. Brain Commun. 2020;2(2):fcaa121. 33094283 10.1093/braincomms/fcaa121 PMC7566327

[16] Ng Kee Kwong KC, Harbham PK, Selvaraj BT, et al 40 years of CSF toxicity studies in ALS: What have we learnt about ALS pathophysiology? Front Mol Neurosci. 2021;14:647895. 33815058 10.3389/fnmol.2021.647895 PMC8012723

[17] Gomez-Pinedo U, Galan L, Yanez M, et al Histological changes in the rat brain and spinal cord following prolonged intracerebroventricular infusion of cerebrospinal fluid from amyotrophic lateral sclerosis patients are similar to those caused by the disease. Neurologia (Engl Ed). 2018;33(4):211–223. La infusion intracerebroventricular prolongada de liquido cefalorraquideo procedente de pacientes con esclerosis lateral amiotrofica provoca cambios histologicos en el cerebro y la medula espinal de la rata similares a los hallados en la enfermedad. 27570180 10.1016/j.nrl.2016.07.002

[18] Scialo C, Tran TH, Salzano G, et al TDP-43 real-time quaking induced conversion reaction optimization and detection of seeding activity in CSF of amyotrophic lateral sclerosis and frontotemporal dementia patients. Brain Commun. 2020;2(2):fcaa142. 10.1093/braincomms/fcaa142 PMC7566418 33094285

[19] Saborio GP, Permanne B, Soto C. Sensitive detection of pathological prion protein by cyclic amplification of protein misfolding. Nature. 2001;411(6839):810–813. 11459061 10.1038/35081095

[20] Saijo E, Ghetti B, Zanusso G, et al Ultrasensitive and selective detection of 3-repeat tau seeding activity in Pick disease brain and cerebrospinal fluid. Acta Neuropathol. 2017;133(5):751–765. 28293793 10.1007/s00401-017-1692-z

[21] Kraus A, Saijo E, Metrick MA II, et al Seeding selectivity and ultrasensitive detection of tau aggregate conformers of Alzheimer disease. Acta Neuropathol. 2019;137(4):585–598. 30570675 10.1007/s00401-018-1947-3 PMC6426988

[22] Metrick MA II, Ferreira NDC, Saijo E, et al A single ultrasensitive assay for detection and discrimination of tau aggregates of Alzheimer and Pick diseases. Acta Neuropathol Commun. 2020;8(1):22. 32087764 10.1186/s40478-020-0887-z PMC7036215

[23] Garrido A, Fairfoul G, Tolosa ES, Marti MJ, Green A. Barcelona LSG. Alpha-synuclein RT-QuIC in cerebrospinal fluid of LRRK2-linked Parkinson’s disease. Ann Clin Transl Neurol. 2019;6(6):1024–1032. 31211166 10.1002/acn3.772 PMC6562027

[24] Fairfoul G, McGuire LI, Pal S, et al Alpha-synuclein RT-QuIC in the CSF of patients with alpha-synucleinopathies. Ann Clin Transl Neurol. 2016;3(10):812–818. 27752516 10.1002/acn3.338 PMC5048391

[25] Concha-Marambio L, Farris CM, Holguin B, et al Seed amplification assay to diagnose early Parkinson’s and predict dopaminergic deficit progression. Mov Disord. 2021;36(10):2444–2446. 34236720 10.1002/mds.28715 PMC8530949

[26] Russo MJ, Orru CD, Concha-Marambio L, et al High diagnostic performance of independent alpha-synuclein seed amplification assays for detection of early Parkinson’s disease. Acta Neuropathol Commun. 2021;9(1):179. 34742348 10.1186/s40478-021-01282-8 PMC8572469

[27] Afroz T, Chevalier E, Audrain M, et al Immunotherapy targeting the C-terminal domain of TDP-43 decreases neuropathology and confers neuroprotection in mouse models of ALS/FTD. Neurobiol Dis. 2023;179:106050. 36809847 10.1016/j.nbd.2023.106050

[28] Ollier R, Fuchs A, Gauye F, et al Improved antibody pharmacokinetics by disruption of contiguous positive surface potential and charge reduction using alternate human framework. MAbs. 2023;15(1):2232087. 37408314 10.1080/19420862.2023.2232087 PMC10324452

[29] Turner MR, Hardiman O, Benatar M, et al Controversies and priorities in amyotrophic lateral sclerosis. Lancet Neurol. 2013;12(3):310–322. 23415570 10.1016/S1474-4422(13)70036-X PMC4565161

[30] Cordts I, Wachinger A, Scialo C, et al TDP-43 proteinopathy specific biomarker development. Cells. 2023;12(4):597. 36831264 10.3390/cells12040597 PMC9954136

[31] Maniecka Z, Polymenidou M. From nucleation to widespread propagation: A prion-like concept for ALS. Virus Res. 2015;207:94–105. 25656065 10.1016/j.virusres.2014.12.032

[32] Kasai T, Kojima Y, Ohmichi T, et al Combined use of CSF NfL and CSF TDP-43 improves diagnostic performance in ALS. Ann Clin Transl Neurol. 2019;6(12):2489–2502. 31742901 10.1002/acn3.50943 PMC6917342

[33] Kumar ST, Nazarov S, Porta S, et al Seeding the aggregation of TDP-43 requires post-fibrillization proteolytic cleavage. Nat Neurosci. 2023;26(6):983–996. 37248338 10.1038/s41593-023-01341-4 PMC10244175

[34] Stine WB, Jungbauer L, Yu C, LaDu MJ. Preparing synthetic abeta in different aggregation states. Methods Mol Biol. 2011;670:13–32. 20967580 10.1007/978-1-60761-744-0_2 PMC3752843

[35] Jankovic J, Goodman I, Safirstein B, et al Safety and tolerability of multiple ascending doses of PRX002/RG7935, an anti-alpha-synuclein monoclonal antibody, in patients with Parkinson disease: A randomized clinical trial. JAMA Neurol. 2018;75(10):1206–1214. 29913017 10.1001/jamaneurol.2018.1487 PMC6233845

[36] Schofield DJ, Irving L, Calo L, et al Preclinical development of a high affinity alpha-synuclein antibody, MEDI1341, that can enter the brain, sequester extracellular alpha-synuclein and attenuate alpha-synuclein spreading in vivo. Neurobiol Dis. 2019;132:104582. 31445162 10.1016/j.nbd.2019.104582

[37] Kouhi A, Pachipulusu V, Kapenstein T, Hu P, Epstein AL, Khawli LA. Brain disposition of antibody-based therapeutics: Dogma, approaches and perspectives. Int J Mol Sci. 2021;22(12):6442. 34208575 10.3390/ijms22126442 PMC8235515

[38] Mirian A, Moszczynski A, Soleimani S, Aubert I, Zinman L, Abrahao A. Breached barriers: A scoping review of blood-central nervous system barrier pathology in amyotrophic lateral sclerosis. Front Cell Neurosci. 2022;16:851563. 35431812 10.3389/fncel.2022.851563 PMC9009245

[39] Alarcan H, Al Ojaimi Y, Lanznaster D, et al Taking advantages of blood-brain or spinal cord barrier alterations or restoring them to optimize therapy in ALS? J Pers Med. 2022;12(7):1071. 35887567 10.3390/jpm12071071 PMC9319288

[40] Iwasaki K, Uno Y, Utoh M, Yamazaki H. Importance of cynomolgus monkeys in development of monoclonal antibody drugs. Drug Metab Pharmacokinet. 2019;34(1):55–63. 29655914 10.1016/j.dmpk.2018.02.003

[41] Sims JR, Zimmer JA, Evans CD, et al Donanemab in early symptomatic Alzheimer disease: The TRAILBLAZER-ALZ 2 randomized clinical trial. JAMA. 2023;330(6):512–527. 37459141 10.1001/jama.2023.13239 PMC10352931

[42] Choudhury P, Saroya KK, Anand S, et al Unjumbling the TWIST score for testicular torsion: Systematic review and meta-analysis. Pediatr Surg Int. 2023;39(1):137. 36811717 10.1007/s00383-023-05401-5

[43] Gasset-Rosa F, Lu S, Yu H, et al Cytoplasmic TDP-43 De-mixing independent of stress granules drives inhibition of nuclear import, loss of nuclear TDP-43, and cell death. Neuron. 2019;102(2):339–357.e7. 30853299 10.1016/j.neuron.2019.02.038 PMC6548321

[44] Pozzi S, Codron P, Soucy G, et al Monoclonal full-length antibody against TAR DNA binding protein 43 reduces related proteinopathy in neurons. JCI Insight. 2020;5(21). 10.1172/jci.insight.140420 PMC7710295 33021970

[45] Riemenschneider H, Simonetti F, Sheth U, et al Targeting the glycine-rich domain of TDP-43 with antibodies prevents its aggregation in vitro and reduces neurofilament levels in vivo. Acta Neuropathol Commun. 2023;11(1):112. 37434215 10.1186/s40478-023-01592-z PMC10334564

[46] Arseni D, Hasegawa M, Murzin AG, et al Structure of pathological TDP-43 filaments from ALS with FTLD. Nature. 2022;601(7891):139–143. 34880495 10.1038/s41586-021-04199-3 PMC7612255

[47] Guenther EL, Cao Q, Trinh H, et al Atomic structures of TDP-43 LCD segments and insights into reversible or pathogenic aggregation. Nat Struct Mol Biol. 2018;25(6):463–471. 29786080 10.1038/s41594-018-0064-2 PMC5990464

