# Supplementary material for: Targeting amyotrophic lateral sclerosis by neutralizing seeding-competent TDP-43 in CSF
Source: Brain Commun. 2023 Nov 3;5(6):fcad306. doi: 10.1093/braincomms/fcad306 (PMC10644982; doi:10.1093/braincomms/fcad306)
Supplement: fcad306_Supplementary_Data [file fcad306_supplementary_data.pdf]

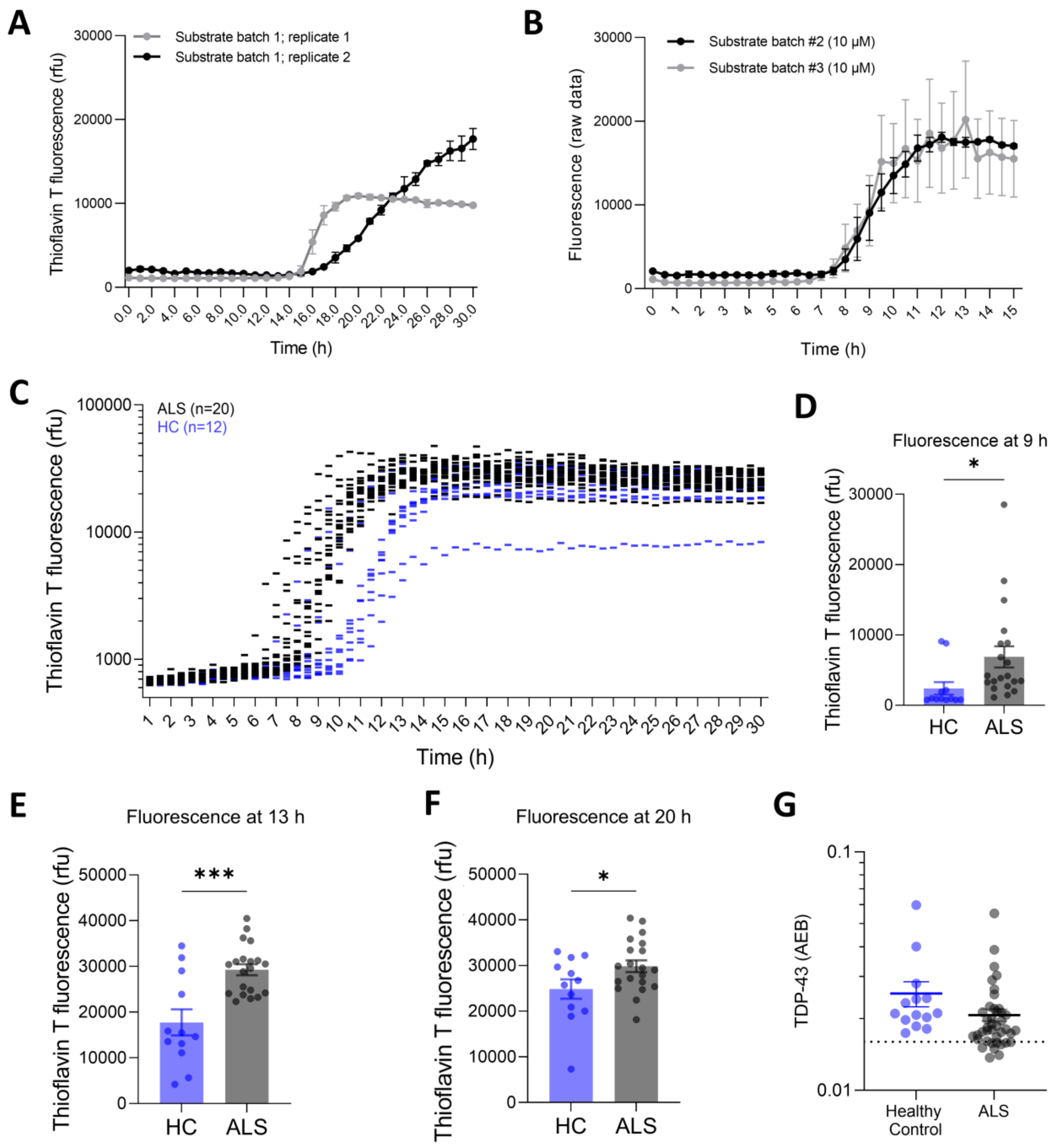

Supplementary Fig. 1: Differentiating ALS patients from HC using TDP-43 SAA

(A-B) Aggregation kinetics of different batches of peptide in absence (A) and in presence (B) of hexafluoro-2-propanol.

C) Aggregation kinetics of reaction substrate in presence of CSF from apparently sporadic ALS patients (sALS; n=20, black dashes) and aged-match healthy controls (HC; n=12, blue dashes) used as seeds in the SAA assay. For each data point, two technical replicates were measured and shown.

(D-E) Quantification of the ThT fluorescence at arbitrary time points 9h (D), 13h (E) and 20h (F). Data shown as mean ± standard error. Unpaired Student's t-test (two-tailed), *p<0.05; ***p<0.001.

G) Average enzymes per bead (AEB) on the y-axis reflecting the levels of TDP-43 in CSF samples from ALS patients (n=41) and HC (n=14) using the in-house established TDP-43 SIMOA® assay. Lower limit of detection is shown by dotted line. Data shown as mean ± standard error.

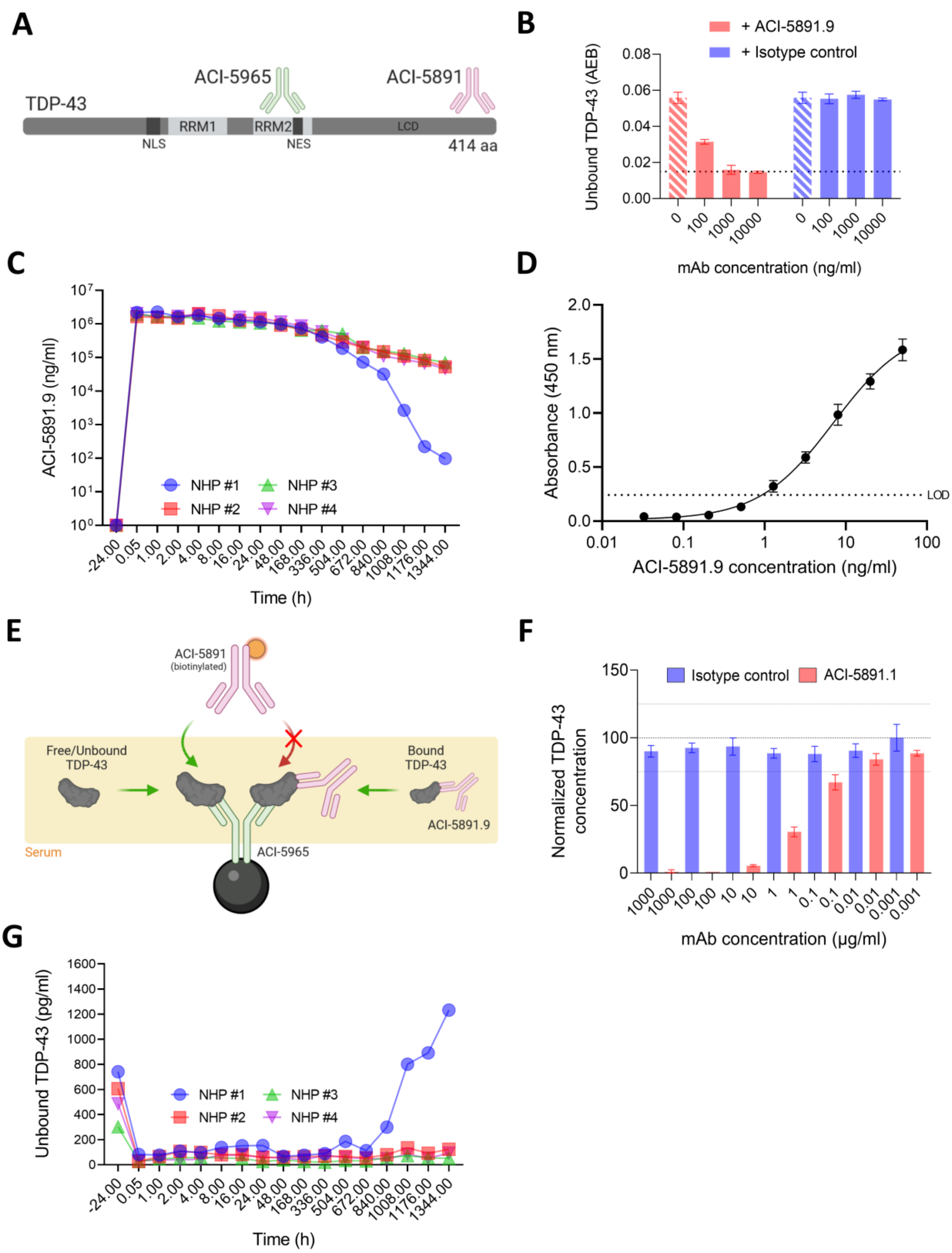

Supplementary Fig. 2: Evaluation of pharmacologically relevant ACI-5891.9 concentrations to saturate TDP-43 in human CSF

(A) Representation of ACI-5965 and ACI-5891 binding sites on TDP-43. RRM1: RNA Recognition Motif 1; RRM2: RNA Recognition Motif 2; LCD: Low-Complexity Domain; NLS: Nuclear Localization Signal; NES: Nuclear Export Sequence.

(B) Detection of free/unbound TDP-43 in human CSF samples pre-incubated with different concentrations of ACI-5891.9 or isotype control for 1 h.

(C) PK profile of ACI-5891.9 in NHP serum upon single intravenous injection at 40 mg/kg, n=4. This representation of the PK profile (x-axis) was intentionally used for comparison with panel G. Presence of anti-drug antibodies (ADA) was confirmed for the NHP #1.

(D) Standard curve for ACI-5891.9 obtained in CSF of non-human primates.

(E) Novel assay design to assess ACI-5891.9 target engagement via measuring free TDP-43 in NHP serum. ACI-5965 is used as capture antibody after coating to magnetic beads. A biotinylated version of ACI-5891 is used as detection antibody.

(F) Evaluation of the interference between isotype control or humanized ACI-5891 with the ACI-5891-biotinylated detection mAb in NHP serum. Several concentrations of either isotype control or humanized ACI-5891 (from 0.001 to 1000 µg/mL) were incubated in a naïve NHP serum and a complete interference was observed with 10, 100 and 1000 µg/mL of humanized ACI-5891. Data shown as ratio of extrapolated recombinant TDP-43 concentrations of samples over control pre-incubated with PBS.

(G) Extrapolated free TDP-43 concentration shown on the y-axis for serum samples from NHPs following single-dose, intravenous administration at 40 mg/kg. A strong decrease in free TDP-43 (or increase in bound TDP-43) was observed from 3 min post-dosing with ACI-5891.9 indicating a fast and effective target engagement.

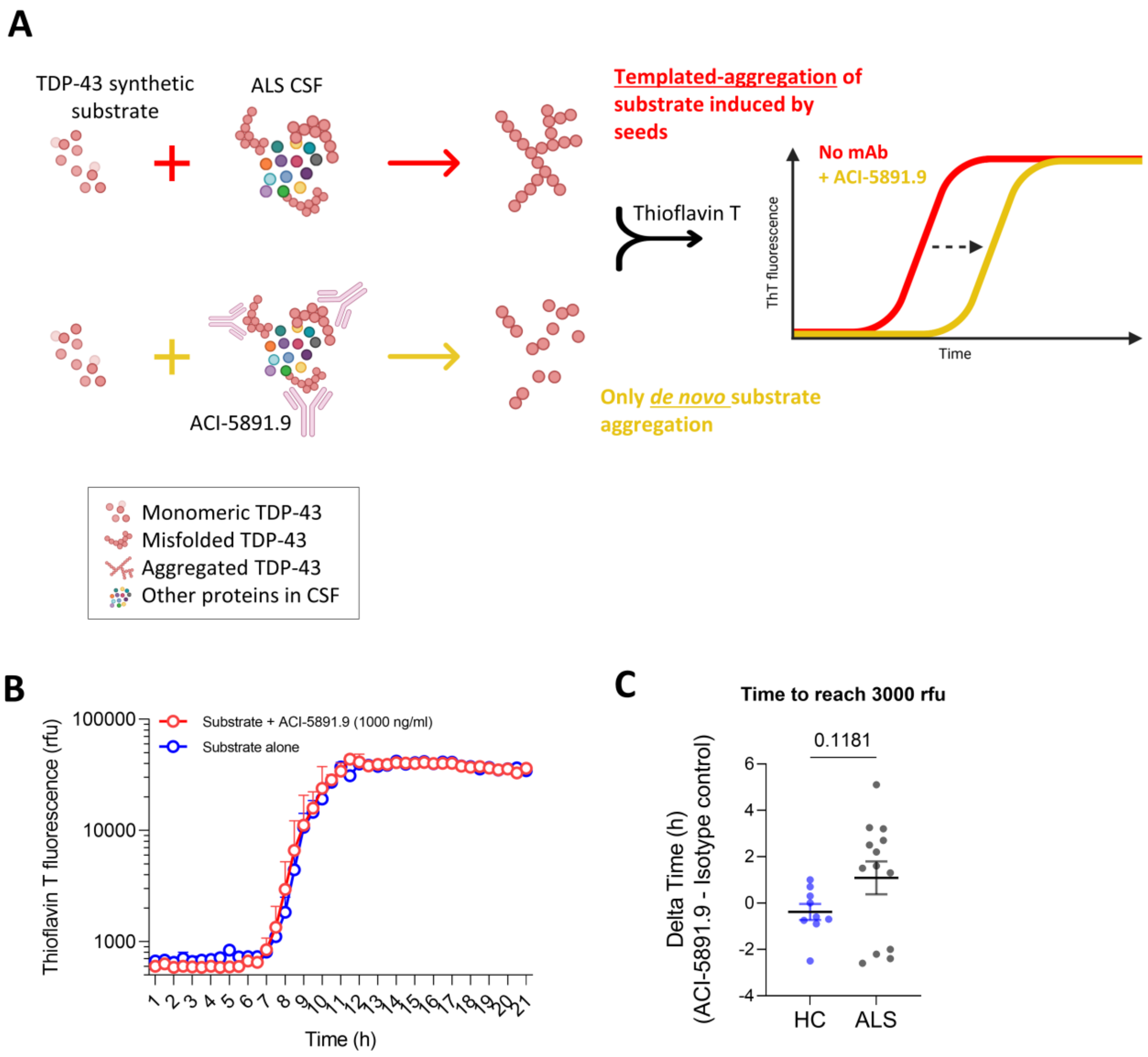

Supplementary Fig. 3: ACI-5891.9 neutralizes TDP-43 seeding species in CSF of ALS patients

(A) Graphical summary of SAA assessing neutralization of TDP-43 seeds by ACI-5891.9.

(B) Aggregation kinetics of the seed-free substrate (10 µM) in presence of 1000 ng/ml (0.006 µM) ACI-5891.9.

(C) Difference of the time needed to reach 3000 rfu when CSF samples were pre-incubated with ACI-5891.9 versus isotype control in SAA for HC (n=10) and ALS (n=14) patient samples. Data shown as mean ± standard error. An unpaired Student's t-test (two-tailed) was used.

Parameters	Unit	ACI-5891.9
Cl	mL/h/kg	0.083
V_c	mL/kg	23.3
Q1	mL/h/kg	0.4
V_{p1}	mL/kg	12.3
$T_{1/2\ b}$	days	12.7

Supplementary table 1: Population PK parameter estimates after single intravenous administration of 40 mg/kg in NHPs

Cl: clearance from the central compartment, Vc: volume of the central compartment, Q1: inter-compartment distribution clearance, Vp1: volume of the peripheral compartment. T1/2 b: terminal half-life.
